# White matter deficits assessed by diffusion tensor imaging and cognitive dysfunction in psychostimulant users with comorbid human immunodeficiency virus infection

**DOI:** 10.1186/s13104-015-1501-5

**Published:** 2015-09-30

**Authors:** Victor M. Tang, Donna J. Lang, Chantelle J. Giesbrecht, William J. Panenka, Taylor Willi, Ric M. Procyshyn, Fidel Vila-Rodriguez, Willough Jenkins, Tania Lecomte, Heidi N. Boyda, Ana Aleksic, G. William MacEwan, William G. Honer, Alasdair M. Barr

**Affiliations:** Department of Psychiatry, University of British Columbia, 2255 Wesbrook Mall, Vancouver, V6T2A1 Canada; Department of Radiology, University of British Columbia, 3350-950 W 10th Avenue, Vancouver, V5Z1M9 Canada; British Columbia Mental Health & Addictions Research Institute, 938 W 28th Avenue, Vancouver, V5Z4H4 Canada; Department of Psychology, Simon Fraser University, 8888 University Drive, Burnaby, V5A1S6 Canada; Département de Psychologie, Université de Montréal, Montreal, QC Canada; Department of Pharmacology, University of British Columbia, 2176 Health Sciences Mall, Vancouver, BC V6T1Z3 Canada

**Keywords:** Comorbidity, Diffusion tensor imaging, Human immunodeficiency virus, Magnetic resonance imaging, Neurocognitive, Psychostimulant

## Abstract

**Background:**

Psychostimulant drug use is commonly associated with drug-related infection, including the human immunodeficiency virus (HIV). Both psychostimulant use and HIV infection are known to damage brain white matter and impair cognition. To date, no study has examined white matter integrity using magnetic resonance imaging (MRI) diffusion tensor imaging (DTI) in chronic psychostimulant users with comorbid HIV infection, and determined the relationship of white matter integrity to cognitive function.

**Methods:**

Twenty-one subjects (mean age 37.5 ± 9.0 years) with a history of heavy psychostimulant use and HIV infection (8.7 ± 4.3 years) and 22 matched controls were scanned on a 3T MRI. Fractional anisotropy (FA) values were calculated with DTI software. Four regions of interest were manually segmented, including the genu of the corpus callosum, left and right anterior limbs of the internal capsule, and the anterior commissure. Subjects also completed a neurocognitive battery and questionnaires about physical and mental health.

**Results:**

The psychostimulant using, HIV positive group displayed decreased white matter integrity, with significantly lower FA values for all white matter tracts (p < 0.05). This group also exhibited decreased cognitive performance on tasks that assessed cognitive set-shifting, fine motor speed and verbal memory. FA values for the white matter tracts correlated with cognitive performance on many of the neurocognitive tests.

**Conclusions:**

White matter integrity was thus impaired in subjects with psychostimulant use and comorbid HIV infection, which predicted worsened cognitive performance on a range of tests. Further study on this medical comorbidity is required.

## Background

Illicit drug use represents a pervasive public health concern, creating substantial cost and burden for individuals and society. Psychostimulant drugs, including cocaine, amphetamines and methamphetamine are among the most commonly used substances worldwide [1]. Concern about the widespread use of psychostimulants includes the health risks associated with chronic substance use [2, 3].

Research with magnetic resonance imaging (MRI) has helped to elucidate the deleterious effects of psychostimulant use on brain structure, with consistent abnormalities being found within fronto-striatal systems [4]. Diffusion tensor imaging (DTI) has been used to examine the effects of psychostimulant use on the white matter tracts of the brain. DTI measures the directional coherence of the diffusion of water in tissue. A reduction in anisotropy indicates reduced organization or integrity of white matter, usually as a result of axonal compromise, or increased membrane permeability (e.g. demyelination) [5]. The most common method for quantifying anisotropy is through fractional anisotropy (FA). Researchers have reported decreased FA in frontal white matter in methamphetamine users [6, 7] and in frontal white matter and the corpus callosum of cocaine users [8, 9] (Table 1). Decreases have also been found in major white matter tracts connecting the right and left hemispheres, with decreased FA in the genu of the corpus callosum associated with methamphetamine use [10, 11] and cocaine use [9, 12].

**Table 1:** Diffusion tensor imaging studies in stimulant abuse

References	n (drug users, controls)	Stimulant	Results^a^	Exclusions^b^
Moeller et al. [12]	18, 18	Cocaine	Decreased FA in genu and rostrum of CC, no difference in other areas of CC	Nonpsychiatric medical disorders which affect the CNS
Lim et al. [58]	21, 21	Cocaine	Decreased FA in inferior frontal cortex, but not superior frontal or occipital cortex	–
Ma et al. [8]	19, 18	Cocaine	Decreased FA in isthmus and splenium of the CC, increased RD in isthmus and rostral body, increased MD in isthmus and rostral body	Medical disorders that may affect the CNS
Lane et al. [9]	15, 18	Cocaine	Decreased FA in parietal and frontal regions (mainly right corticospinal tract, right superior corona radiata, bilateral anterior corona radiata) and anterior CC, increased RD in frontal, parietal, and occipital regions (mainly bilateral corticospinal tracts, superior corona radiata, left posterior corona radiata, left optic radiation, left superior longitudinal fasciculus, left posterior thalamic radiation, left retrolenticular internal capsule) and left posterior body of CC and bilateral anterior CC	Medical disorders that may affect the CNS, comorbid alcohol dependence
Bell et al. [59]	43, 43	Cocaine	Decreased FA in left anterior callosal fibers, left genu of the CC, right superior longitudinal fasciculus, right callosal fibers, and the superior corona radiata bilaterally	Diagnosis of HIV
Ma et al. [60]	12, 12	Cocaine	Decreased FA in bilateral anterior corona radiata, but not in the CC	Medical disorders that may affect the CNS
Chung et al. [61]	32, 30	Methamphetamine	Decreased FA bilateral frontal white matter at AC-PC plane, right frontal above AC-PC plane	Lifetime significant medical illnesses
Alicata et al. [62]	30, 30	Methamphetamine	Decreased FA right frontal white matter; higher ADC left caudate and bilateral putamen	Comorbid medical illnesses, no alcohol or other drug dependence
Kim et al. [10]	11, 13	Methamphetamine	Decreased FA in genu of CC	No screening for HIV status, alcohol dependence, lifetime use of other addictive drug
Salo et al. [45]	37, 17	Methamphetamine	No significant difference in FA or ADC of CC	Substance dependence other than methamphetamine in the past year, alcohol abuse in past 5 years, HIV seropositivity
Tobias et al. [11]	23, 18	Methamphetamine	Decreased FA in right prefrontal cortex superior to AC-PC, genu of CC, mid-caudal superior corona radiata bilaterally, right perforant fibers (hippocampus)	Heavy marijuana or alcohol use, HIV seropositivity
Lin et al. [63]	18, 22	Methamphetamine	No significant difference in FA, RD, MD, or AD for caudate and putamen	Dependence on any other drug except nicotine requiring medical treatment

– Information not provided, *AC-PC* anterior commissure-posterior commissure, *ADC* apparent diffusion coefficient, *AD* axial diffusivity, *CC* corpus callosum, *FA* fractional anisotropy, *MD* mean diffusivity, *RD* radial diffusivity

^a^Drug users compared to healthy controls

^b^Only exclusion criteria relevant to drug use or HIV/AIDS

When studying the effects of drug use on the brain, it is also important to consider the clinically complex issue of comorbidity that is common in many psychostimulant users. The incidence of infectious disease in psychostimulant users is high, including the human immunodeficiency virus (HIV) [13] that may be acquired by intravenous drug use and unsafe sexual practices [14]. In HIV/AIDS affected individuals who are not psychostimulant abusers, several brain regions have been shown to exhibit decreased FA values [15–18] (Table 2). White matter changes are related to duration of infection, and are more affected in those with cognitive deficits [19]. Cognitive impairment is seen in as much as 63 % of this population [20] and such deficits correlate with FA value decreases [21–23].

**Table 2:** Diffusion tensor imaging studies in HIV/AIDS

References	N (HIV ± , controls)	Disease duration	On anti-retroviral therapy (%)	CD4 count (mean cells/uL)	Results^a^	Exclusions^b^
Pomara et al. [64]	6, 9	–	83 %	288.67	Decreased FA in frontal lobes. No difference in FA for parietal lobe, temporal lobe, or genu or splenium of CC. Increased FA in the posterior limb of the internal capsule, no difference in anterior limb of internal capsule. No differences in MD	Current alcohol or substance abuse
Ragin et al. [65]	11, 11	–	100 %	–	No differences in centrum semiovale, caudate, putamen	None relevant
Thurnher et al. [66]	60, 30	–	–	–	Decreased FA in genu of CC, increased ADC in genu of the CC	None relevant
Wu et al. [67]	11, 11^a^ same sample as Ragin et al. [82]	–	100 %	–	Decreased FA in splenium of CC. Increased MD in splenium of CC. No differences in genu of CC and frontal white matter	None relevant
Pfefferbaum et al. [68]	42, 88	Mean 8.4 years	79 %	546.6	No significant differences in CC regions	Recent drug abuse or dependence
Stebbins et al. [69]	30, 30	Mean 9.9 years	77 %	612.8	Decreased FA in right middle frontal gyrus, left cuneus, left precuneus, right precentral gyrus, right cingulum, right insula, right internal capsule near pulvinar, increased FA in bilateral medial frontal lobes, bilateral middle frontal gyrus, right interior frontal lobe, left precentral gyrus, right cingulum, right parietal lobes. Decreased MD in right middle frontal gyrus and right ventriculus lateralis. Increased MD in left superior frontal, bilateral middle frontal gyrus, right cingulum, bilateral precentral gyrus, right superior temporal gyrus, left middle temporal gyrus, left cuneus, right anterior and posterior limbs of the internal capsule	Substance abuse within the last 6 months
Chang et al. [37]	39, 32	Mean 13.7 years	–	461.9	Decreased FA in parietal white matter and increased MD in frontal white matter, after 1 year follow up increased MD in frontal and parietal white matter, putamen, and genu of CC	History of drug dependence in the past
Schulte et al. [70]	19, 17	–	68.40 %	486	No changes in FA and MD of CC	Axis I psychiatric diagnosis
Chen et al. [39]	29, 18	–	62 %	–	Decreased FA in frontal, parietal, temporal, occipital white matter and CC in HIV associated dementia subgroup, and in HIV non-dementia subgroup decreased in frontal, occipital white matter and CC. Increased MD in both dementia and non-dementia in frontal, parietal, temporal and CC white matter. Increased AD in parietal white matter and CC in dementia subgroup, and increase in CC in non dementia subgroup. Increase in RD in frontal, parietal, temporal, occipital white matter and CC in dementia while non dementia incrase in frontal, parietal, temporal white matter and CC. No differences in any measures for the internal capsule	–
Gongvatana et al. [22]	39, 25	–	82 %	529	Decreased FA in posterior limb of the internal capsule, right interior longitudinal fasciculus, right optic radiation. No significant differnce in MD	Substance use disorder in last 6 months
Pfefferbaum et al. [71]	42, 88^a^ same sample as in Pfefferbaum et al. [68]	Mean 8.4 years	79 %	546.6	No changes in FA in internal capsule, external capsule, fornix, frontal forceps, occipital forceps, superior cingulate, inferior cingulate, superior longitudinal fasciculus, inferior longitudinal fasciculus, pontocerebellar tract, cerebellar hemispheres, CC. Increased longitudinal diffusivity in internal capsule and superior cingulate	Alcohol or substance abuse
Muller-Oehring et al. [72]	21, 19	–	86 %	519	No changes in FA of CC	–
Hoare et al. [73]	46, 10	Diagnosis made in last 6 months	0 %	211.51	Decreased FA in rostrum of CC, sagittal striatum, and the cingulum	Recent substance abuse history within 6 months
Du et al. [74]	10, 24	–	–		Decreased FA and increased MD for whole brain white matter	none relevant
Jacqueline et al. [18]	40, 10	–	0 %	193.61	In subgroup with poor prospective memory (n = 27), there was decreased FA in the corpus callosum, sagittal striatum, and superior longitudinal fasciculus. In subgroup with good prospective memory (n = 13), there was increased FA in superior longitudinal fasciculus. No differences in MD	Recent 6 month drug abuse history
Schulte et al. [75]	16, 15	–	81 %	556.3	Decreased FA in inferior longitudinal fasciculus and uncinate fasciculus	Non alcohol drug abuse or dependence in last 3 months or use of drugs in the past month
Stubbe-Drager et al. [76]	19, 19	Mean 6.8 years	68 %	–	Decreased FA in CC, temporal, and posterior region	Current alcohol or substance abuse
Wright et al. [77]	42, 21	Mean 4.5 years in antiretroviral group, 1.5 years in antiretroviral naïve group	50 %	384 in antiretroviral group, 371 in antiretroviral naïve	Antiretroviral naïve (n = 21) had decreased MD, AD, RD for each CC region and the centrum semiovale, no differences in FA. No differences in antiretroviral group (n = 21) in any measure	Active substance abuse
Leite et al. [16]	34, 27	Median 13 years	–	679	Decreased FA in body of CC, no differences in FA of corona radiata. Increased RD and MD in body of CC, left superior corona radiata, left posterior corona radiata. Increased MD in right posterior corona radiata. No significant differences in any measures of right anterior and superior corona radiata and left anterior corona radiata, cingulate gyri	–
Xuan et al. [78]	23, 20	–	–	–	Decreased FA and increased ADC in CC genu and rostrum, body, and splenium, the lateral periventricular white matter, and frontal and parietal lobar white matter, internal capsule, and occipital white matter in the symptomatic group. In the asymptomatic group, significantly decreased FA and ADC in all regions except internal capsule and occipital white matter	None relevant
Zhu et al. [19]	50, 13	Mean 10.7 years in no cognitive impairment group, 15.1 with cognitive impairment	82 %	386.3 no CI, 230.4 with CI	Increased MD in posterior area of frontal, temporal, and parietal lobe for non cognitive impaired, included prefrontal in cognitively impaired, FA decreased only in cognitively impaired subgroup in fibers associated or connected to prefrontal cortex	Abusing drugs or alcohol in last 6 months
Kamat et al. [79]	19, 19	Mean 5.8 years	–	340 (median)	Decreased FA in bilateral anterior corona radiata, genu of CC, and left orbital-medial prefrontal cortex	Current substance dependence of abuse
Nir et al. [80]	56, 31	Mean 20 years	95 %	520	Decreased FA and MD, RD, and AD increases diffusely through whole brain white matter, greatest differences in CC and projection fibers of corona radiata	Major psychiatric illness
Correa et al. [81]	47, 19	Mea 13.06 years (planning deficit group) and 12.50 years (no planning deficit)	100 %	693.41 (planning deficit group) and 606.25 (no planning deficits)	In the subgroup with deficits in planning, decreased FA and increased MD and RD in bilateral anterior thalamic radiations, bilateral inferior fronto-occipital fasciculi, genu and splenium of CC, bilateral superior longitudinal fascicule, bilateral uncinate fasciculi, increased AD in left anterior thalamic radiation, left inferior fronto-occipital, and left longitudinal fasciculi. In the subgroup with no planning deficits, no significant changes in FA, RD, MD, and AD	Illicit drug use in the past year
Ragin et al. [82]	15, 20	Less than 100 days	53 %	580	Decreased FA in the CC and increased MD in caudate	Chronic or active drug abuse
Wright et al. [83]	78, 19	<1 year for primary infection group (n = 62) and mean 11.5 years for chronic infected group (n = 16)	0 %	573 for primary infected group, 223 for chronic infected group	Decreased FA and increased MD for CC and whole brain white matter in chronically infected subgroup (n = 16) but not primary infected group of < 1 year (n = 62)	–

– Information not provided, *AC-PC* anterior commissure-posterior commissure, *ADC* apparent diffusion coefficient, *AD* axial diffusivity, *CC* corpus callosum, *FA* fractional anisotropy, *MD* mean diffusivity, *RD* radial diffusivity

^a^Drug users compared to healthy controls

^b^Only exclusion criteria relevant to drug use or HIV/AIDS

In the present study, our goal was to examine the neuroanatomical and cognitive changes in psychostimulant users with comorbid HIV infection, using DTI. To our knowledge, there has not been a study of FA values in this comorbid population, even though there known effects of psychostimulant use and HIV on white matter integrity. We have recently reported that comorbid addiction and infectious disease can significantly impact factors such as everyday functioning [24, 25]. In the present study, we have focused specifically on the key frontotemporal white matter pathways of the genu of the corpus callosum, the right and left anterior limbs of the internal capsule, and the anterior commissure. It has been proposed that forebrain regions that are connected by these tracts are most affected by psychostimulant use, as they receive dopamine projections from the midbrain, which are a key substrate of the toxic effects of psychostimulant exposure [2], and are important for neurocognitive function. Participants were also subject to a comprehensive neuropsychological test battery in order to assess function in multiple cognitive domains.

## Methods

### Participants

Twenty-one HIV-positive subjects seeking care for psychostimulant use (the “patient” group) were recruited from St. Paul’s and Vancouver General Hospital in Vancouver, Canada. Referrals were through staff clinicians in the emergency department. Patients were ≥19 years old, had a history of psychostimulant exposure, HIV-positive and could provide informed consent. Twenty-two age, gender and premorbid IQ matched controls were recruited from the same geographical community. All subjects in the study provided written consent. The study was approved by the UBC Clinical Research Ethics Board (CREB), in accordance with Canadian tri-council guidelines and the Declaration of Helsinki. Exclusion criteria included: surgery in previous 6 months, pregnancy, metal implants, claustrophobia, uncorrectable visual impairment and IQ <70.

### MRI acquisition

A 3Tesla MRI (Philips System) with an eight channel head coil was used. A T1-weighted 3D fast spoiled gradient echo (SPGR) inversion recovery prepped series for volumetric assessment was performed for region of interest (ROI) seeding in structural space in the transverse plane with: TR = 7.7 ms, TE = 3.6 ms, flip angle = 8°, FOV = 256 × 170 × 200 mm, acquisition and reconstruction matrices = 256 × 256, 170 slices, slice thickness = 1 mm, gap = 0, 1 mm isotropic voxel, SENSE = 0. DTI images were acquired in the transverse plane with TR = 7500 ms, TE = 54 ms, b-value = 800, 16 directions, flip angle = 90°, acquisition matrix = 112 × 112, reconstruction matrix = 110 × 110, voxel dimensions = 0.875 mm × 0.875 mm × 2, FOV = 224 × 144 × 224 mm, 72 slices, slice thickness = 2 mm, gap = 0, SENSE = 1.

### ROI manual segmentation

The rostrum and genu of the corpus callosum, the right and left anterior limb of the internal capsule, and the anterior commissure were manually segmented on the T1-SPGR structural scans using the interactive masking tool from FMRIB Software Library 4.1. The anterior limb of the internal capsule was well visualized as a visible white matter tract bounded medially by the caudate and laterally by the putamen on the axial plane, seven slices superior to the first visualization of the anterior commissure.

The anterior commissure was manually segmented axially. The analysis of this ROI was restricted to fibers that crossed two geometrically placed ROIs 4 mm lateral on each side of a mid-sagittal line drawn in the coronal plane. Borders of the corpus callosum genu were traced on the mid-sagittal slice in the sagittal plane. The posterior border was defined with a vertical line marked by the anterior-most point of the inner convexity of the anterior callosum. To remove extraneous fibers and restrict analysis to fronto-temporal connections, additional ROIs from automatically segmented masks were applied to restrict fiber analysis to target tracts. For the genu, all other regions of the corpus callosum were excluded, as well as the fornix. An occipital lobe mask excluded fibers to the corpus callosum genu, anterior limb of the internal capsule, and anterior commissure. Intra-rater reliabilities for FA values for all ROIs. Manual ROI selection was repeated on a random set of five controls and five patients for each tract. Intra-class correlation coefficients were 0.83–0.96 for all tracts.

### Image processing

Image processing was performed on an AppleMacPro Tower Quad Core Intel computer, OS X version 10.5.8. Raw images were converted to NIFTI format with MICRON dcm2nii shareware. Processing of diffusion weighted data for VBM-style analysis, specifically motion and standard eddy current distortion correction as recommended by FSL (see FRMRIB-FDT v2.0 pipeline), local modeling of diffusion parameters, spatial registration, tractography and local fitting of diffusion tensors was performed with FMRIB Diffusion Toolbox as part of the FMRIB Software Library (FSL v4.1.4 http://www.fmrib.ox.ax.uk/fsl). DTI data were registered from diffusion space to structural space and from structural space to standard (MN152) space. Prior to analysis, all diffusion scans were reconstructed to a voxel dimension of 0.88 × 0.88 × 2.0 to mitigate partial volume effects. Tracts were generated from the manual ROI seeds. All tracts were subsequently normalized by seed size. The minimum probability threshold for normalized tracts was set to 0.2 to avoid over-inclusion of non-tract voxels. Volume extraction of frontal pathways from FSL was based on standardized automated reconstruction from the Bayesian framework, in which probabilistic estimation from the diffusion images is calculated based on multiple compartments of anisotropic diffusion and a single compartment of isotropic diffusion per voxel. This is expressed as the diffusion data at any given individual voxel as a function of volume and orientation of any given compartment. FSL applies automated summation of the voxel volumes associated with the manually placed tractography seed at the recommended threshold to calculate the final tract volume. One scan had to be excluded due to motion artifact. All scans were reviewed by a neuroradiologist for evidence of brain injury which could have affected DTI indices; no scans were required to be excluded from the analysis.

Volumetric segmentation was performed with Freesurfer. Processing included motion correction, removal of non-brain tissue, automated Talairach transformation, segmentation of the subcortical white matter and deep gray matter volumetric structures, intensity normalization, tessellation of the gray/white matter boundary, automated topology correction, and surface deformation via intensity gradients [26]. Maps were created using spatial intensity gradients across tissue classes and are therefore not simply reliant on absolute signal intensity.

### Cognition

Before testing, subjects were asked if they were currently experiencing the effects of a drug. If they answered affirmatively, or the research assistant suspected them of having just used a drug, they were excluded from the study. Premorbid intellectual functioning was estimated from the Full Scale IQ score on the North American adult reading test (NAART). All subjects completed a comprehensive neuropsychological battery. Multiple cognitive domains were evaluated (neurocognitive tests and outcome variables listed in parentheses); including processing/visual scanning speed and cognitive set-shifting (trail making test; TMT; Trail A and B completion time), fine motor speed and dexterity (Grooved Pegboard; dominant and non-dominant hand completion time), verbal learning and memory (California verbal learning test-II; CVLT-II; total recall trials 1–5, short delay free recall, long delay free recall, and total recognition discriminability).

The computerized Cambridge neuropsychological test automated battery (CANTAB; [27]) was used to examine sustained attention (rapid visual information processing; RVP; A′ signal detection measure of sensitivity), and components of attentional set-shifting (intra/extra dimensional shift; IED; concept formation errors and concept switching errors). Failure to complete a particular stage on the IED subtest ended the task. Therefore, adjusted error scores for all non-completers were established by adding one error to the error total observed in the participant who made the most errors yet completed the given stage.

Score distributions were examined for normality and outliers. For several variables (e.g., Trails A and B, Grooved Pegboard, and RVP A′) outliers were identified, and adjustments to these scores were made according to [28]. To control for the effects of years of education, which differed significantly between groups, unstandardized residuals were calculated by regressing raw cognitive scores for the entire sample on education. For consistency, the signs of unstandardized residuals for certain variables (e.g., errors or speed) were reversed (i.e., multiplied by −1) so that lower values represented worse performance.

### Demographics

Subjects provided demographic information. Drug use over the previous 30 days was collected through the Maudsley Addiction Profile [29] for both the patient group and the control group. A cumulative total of days in the month spent using psychostimulant drugs was also determined, whereby each day spent using a specific psychostimulant drug was added together. A urine drug screen also was obtained for both groups which detected recent use of amphetamines, barbiturates, benzodiazepines, cocaine (including crack), marijuana, methadone, 3,4-methylenedioxymethamphetamine, opiates and tricyclic antidepressants. Confirmation of HIV status was obtained from patient medical charts. For subjects with HIV, an additional questionnaire was administered to gather information about their illness. This is a 21 item questionnaire that includes information about time of infection, past and current treatment, related medical conditions and hospitalization. Psychiatric health was assessed with the Mini International Neuropsychiatric Interview (MINI), which is a well validated structured clinical interview that assesses lifetime mental health and substance use disorders based on Diagnostic and Statistical Manual, 4th ed. (DSM-IV) and the International Classification of Diseases (ICD)-10 criteria [30]. All subjects were offered the opportunity to complete a complete blood count (CBC) with differential. CD4 and CD8 counts were also measured while HIV positive status was confirmed.

### Statistical analysis

A Pearson Chi square (χ^2^) analysis was performed to compare the groups on categorical demographic variables. Independent t-tests compared mean group FA values and cognitive outcome variables for normally distributed variables. For non-normal cognitive data (TMT B and the IED subtests), the non-parametric Mann–Whitney U test was employed. T-tests were two tailed with statistical significance defined as *p* < 0.05. Effect sizes (ES) for group comparisons were also computed using Cohen’s *d* (corresponding to ES of small = 0.2, medium = 0.5, large = 0.8). Pearson correlations (*r*) assessed associations between FA values and cognitive outcome variables. Analyses were performed using SPSS 16 software (SPSS Inc., USA).

## Results

### Demographic variables

The mean age of the patient group was 37.45 (±9.0) years, range 22–58 years. The mean age of controls was 39.55 (±9.0) years, range 20–57 years. The control group included 13 males and 9 females. The patient group included 14 males and 7 females. Gender distribution did not differ between groups (*p* > 0.46). Subjects did not differ in age (*p* > 0.22) and estimated premorbid intellectual functioning on the NAART test (*p* > 0.16) [99.7 ± 8.8 (patients) vs 103.0 ± 6.8 (controls)]. There was a significant group difference in mean years of completed education (t_(41)_ = 2.79 *p* < 0.01) [10.29 ± 2.63 years (patients) vs 12.05 ± 1.33 years (controls)]. Details on HIV status in the patient group are listed in Table 3.

**Table 3:** HIV status

	Patients (n = 21)
Average duration of HIV infection	8.7 ± 4.3 years
Current status HIV or AIDS	HIV 18 (86 %), AIDS 3 (14 %)
Previous HIV-related hospitalizations (Y/N)	11Y (53 %), 10N (47 %)
Currently treated for HIV(Y/N)	15Y (71 %), 6N (29 %)
If not currently treated, were previously	4Y (66 %), 2N (33 %)

Results of the MINI indicated that 10 subjects in the patient group had a lifetime diagnosis of substance-induced psychosis (versus no controls), 9 a history of depression (versus 5 controls), 2 a history of mania (versus no controls) and 2 a history of PTSD (versus 2 controls). All subjects in the patient group met lifetime criteria for non-alcoholic psychoactive substance use disorder, versus no controls. For bloodwork, 16 of the patient group and 17 of the controls completed the blood work. Subjects in the patient group exhibited significantly lower total white blood cell counts, as well as lower CD4 cell counts, higher CD8 cell counts, and a lower CD4:CD8 ratio (Table 4).

**Table 4:** Results of complete blood count and differential

	Controls (n = 17)	Patients (n = 16)
White blood cell count (giga/L)	6.76 ± 1.81	4.93 ± 1.61**
Helper CD4 (%)	45.35 ± 5.32	28.00 ± 16.04**
Helper CD4 Absolute number (per µL)	874.11 ± 192.32	435.00 ± 310.07**
Suppressor CD8 (5)	24.53 ± 7.53	45.94 ± 19.94**
Suppressor CD8 Absolute number (per µL)	480.59 ± 198.82	836.88 ± 735.46
CD4:CD8 ratio	2.04 ± 0.71	0.96 ± 1.09**

Numbers indicate mean score (±SEM) for either the patient or control group. Significant difference between groups * *p* < 0.05, ** *p* < 0.01

### Drug use

Drug use over the previous 30 days is listed in Table 5 (only substances where more than one subject used the drug are included). The groups differed significantly in alcohol, heroin, cocaine powder, crack cocaine, amphetamines and cannabis use, with alcohol use higher in the controls and every other drug higher in the patient group. The most commonly used drug in the patient group was crack cocaine, followed by cocaine powder and cannabis. While only six subjects in the patient group reported using amphetamines in the previous month, *all* subjects in this group had used some form of psychostimulant drug (amphetamines, cocaine powder or crack cocaine) in the previous 30 days, with psychostimulants being used an average of 32 cumulative “days” per person during this period. The results of the urine drug screen correlated well with self-reported drug use, with 18 of the 21 subjects in the patient group testing positive for recent cocaine or amphetamine use (previous 48 h), and only one subject in the control group testing positive for cocaine.

**Table 5:** Substance use over prior 30 days

	Controls (n = 22)	Patients (n = 21)
Alcohol (# using)	16	7**
Alcohol (# days) [4.2 ± 3.7 units]	4.6 ± 5.7	2.4 ± 6.5
Heroin (# using)	0	7**
Heroin (# days) [0.2 ± 0.15 g]	0	3.4 ± 7.9*
Cocaine powder (# using)	2	13**
Cocaine powder (# days) [0.52 ± 0.76 g]	1	9.4 ± 12.1**
Crack cocaine (# using)	0	19**
Crack cocaine (# days) [0.92 ± 1.65 g]	0	18.3 ± 12.3**
Amphetamines (# using)	0	6**
Amphetamines (# days) [0.38 ± 0.40 g]	0	4.0 ± 8.6*
Cannabis (# using)	4	13**
Cannabis (# days) [0.95 ± 0.68 g]	1.6 ± 5.6	14.3 ± 13.9**

Numbers indicate either number of subjects per group using the drug (# using) or mean number of days per 30 days spent using the drug (# days) ± SEM. Significant difference between groups * *p* < 0.05, ** *p* < 0.01. For number of days using each drug, mean daily intake (± SEM) for both groups for cases who used the drug is included in square parentheses

### Fractional anisotropy

**Fig. 1:**
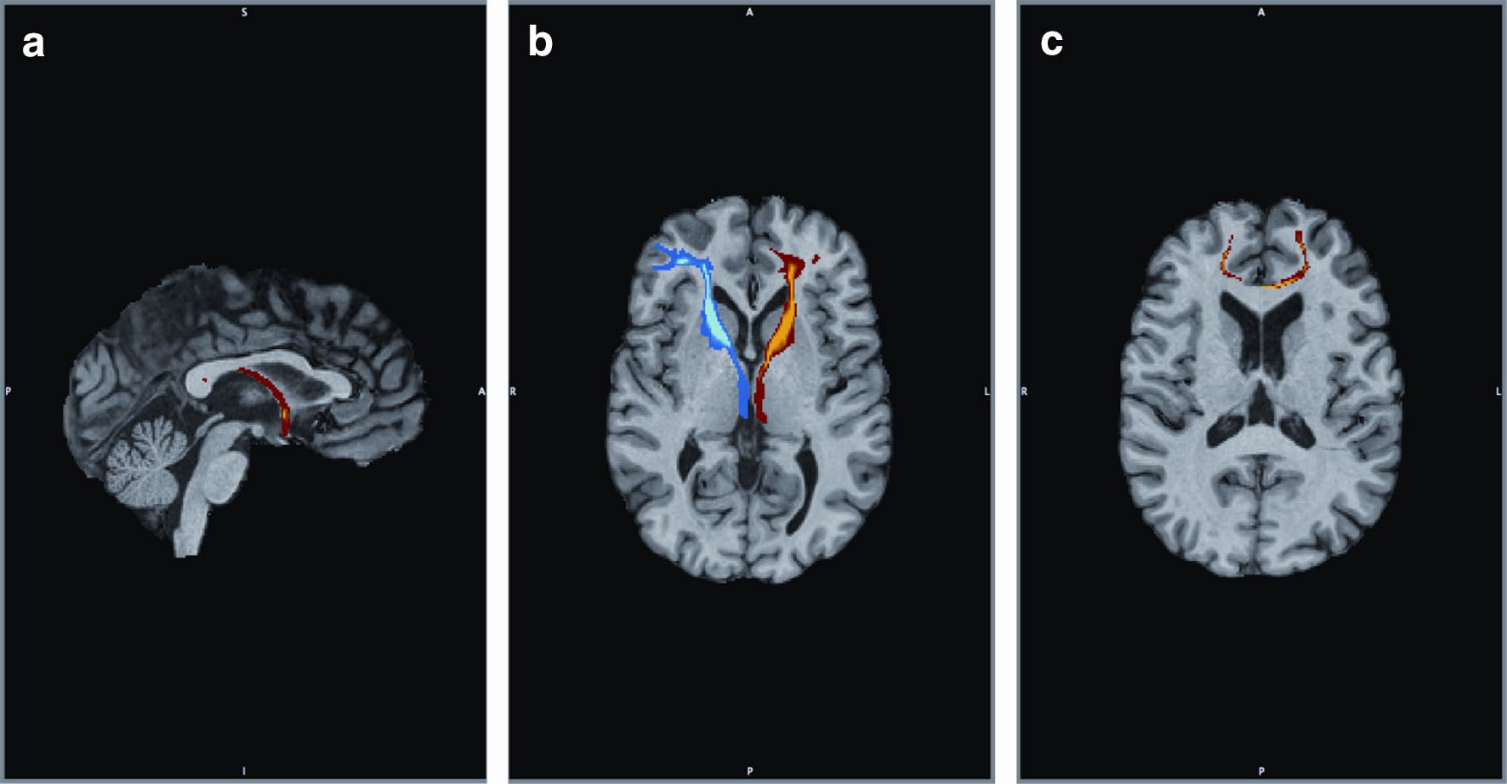
Regions of interest include white matter pathways in the forebrain that are important in neurocognitive function and known to be damaged by HIV infection or psychostimulant use. Pathways include the **a** anterior commissure (*sagittal view*), **b** left (*blue*) and right (*red*) anterior limbs of the internal capsule (*axial view*) and **c** corpus callosum genu (*axial view*)

Summary FA values are shown in Table 6. Significant between-group differences in FA values were seen in the corpus callosum genu (t_(40)_ = 3.84 *p* < 0.001), left (t_(40)_ = 3.23 *p* < 0.005) and right (t_(40)_ = 2.30 *p* < 0.05) anterior limbs of the internal capsule, and in the anterior commissure (t_(40)_ = 2.10 *p* < 0.05), as FA values were lower for the patient group in all of these white matter pathways (Fig. 1). Correlational analyses did not reveal relationships between FA and age in either group for any ROI. There were also significant group differences for medial diffusivity (MD) and radial diffusivity (RD) values for all pathways other than the anterior commissure, and differences in axial diffusivity (AD) values for both the left and right anterior limb of the internal capsule pathways. Total white matter volume in the patient group was smaller than the control group, but this narrowly failed to obtain significance when co-varied for total brain volume (p = 0.052).

**Table 6:** DTI measures of white matter tracts in HIV positive patients with history of psychostimulant use

	Controls (n = 22)	Patients (n = 20)	Effect size (*d*) and *p* value
Genu of CC			
FA	0.3838 ± 0.0254	0.3517 ± 0.0289	1.184; 0.0001*
MD	0.910 ± 0.054	0.951 ± 0.039	−0.864; 0.008*
AD	1.283 ± 0.071	1.301 ± 0.047	−0.296; 0.324
RD	0.723 ± 0.052	0.775 ± 0.046	−1.056; 0.002*
Right ALIC			
FA	0.3555 ± 0.0229	0.3391 ± 0.0233	0.710; 0.027*
MD	0.907 ± 0.064	0.953 ± 0.060	−0.740; 0.022*
AD	1.245 ± 0.071	1.288 ± 0.061	−0.647; 0.044*
RD	0.737 ± 0.064	0.785 ± 0.063	−0.756; 0.020*
Left ALIC			
FA	0.3798 ± 0.0307	0.3539 ± 0.0190	1.002; 0.002*
MD	0.857 ± 0.055	0.914 ± 0.042	−1.157; 0.001*
AD	1.208 ± 0.061	1.254 ± 0.049	−0.827; 0.011*
RD	0.681 ± 0.058	0.745 ± 0.042	−1.254; 0.0001*
AC			
FA	0.3782 ± 0.0499	0.3509 ± 0.0332	0.638; 0.042*
MD	1.107 ± 0.189	1.142 ± 0.200	−0.180; 0.558
AD	1.539 ± 0.187	1.555 ± 0.239	−0.075; 0.773
RD	0.937 ± 0.184	0.893 ± 0.191	0.235; 0.455

Numbers indicate mean score (±SEM) for fractional anisotropy (FA), medial diffusivity (MD), axial diffusivity (AD) and radial diffusivity (RD) values for either the patient or control group; units for MD, AD and RD are (mm^2^/s × 10^−3^). Tracts include the genu of the corpus callosum (CC), left and right anterior limb of the internal capsule (ALIC) and the anterior commissure (AC). Values represent group mean ± SD

* Significant difference between groups p < 0.05

### Neurocognitive performance

The patient group performed worse than controls on all cognitive variables (Table 7). Group differences were seen in cognitive set-shifting (TMT B; *U* = 147.0, *z* = −2.04, *p* < 0.05), fine motor speed and dexterity bilaterally (Grooved Pegboard; dominant hand: t_(41)_ = −3.96, *p* = 0.001; non–dominant hand: t_(41)_ = −3.80, *p* = 0.001), word list acquisition (CVLT-II total recall: t_(41)_ = −2.37, *p* < 0.05) and immediate recall (CVLT-II short delay free recall: t_(41)_ = −2.13, *p* < 0.05).

**Table 7:** Comparison of neurocognitive data between groups

Test-variable	Controls (n = 22)	Patients (n = 21)	Test statistic	Effect size (*d*)
TMT trail A (s)	31.05 ± 1.22	33.81 ± 2.46	−0.74	−0.23
TMT trail B (s)^b^	66.77 ± 4.70	101.29 ± 10.30	147.0 (−2.04)*	−0.65
Grooved pegboard dominant hand (s)	65.86 ± 1.95	95.05 ± 5.49	−3.96**	−1.23
Grooved pegboard non-dominant hand (s)	71.05 ± 2.03	90.35^a^ ± 4.05	−3.80**	−1.21
RVP A′	0.89 ± 0.01	0.86^a^ ± 0.01	−1.84	−0.56
CVLT-II total recall	45.86 ± 1.98	34.33 ± 2.47	−2.37*	−0.72
CVLT-II short delay free recall	10.23 ± 0.74	7.00 ± 0.67	−2.13*	−0.65
CVLT-II long delay free recall	10.45 ± 0.61	7.48 ± 0.79	−1.67	−0.51
CVLT-II recognition discriminability	3.04 ± 0.19	2.47 ± 0.17	−1.30	−0.40
IED EDS errors^b^	9.55 ± 2.16	17.10 ± 2.40	168.0 (−1.53)	−0.61
IED Pre-EDS errors^b^	21.41 ± 4.34	43.86 ± 7.43	191.0 (−0.972)	−0.68

Numbers indicate raw scores of group mean ± SEM with no mathematical transformations (lower scores represent better performance on variables measured in seconds or errors)

Significant difference between groups * *p* < 0.05, ** *p* < 0.01

*TMT* trail making test, *RVP* rapid visual information processing, *CVLT* California verbal learning test, *IED* intra/extra dimensional shift

^a^n = 20; test statistic = *t* value from independent *t*-tests or ^b^ Mann–Whitney U (z) value from the non-parametric Mann–Whitney U test for non-normal data

### Relationship between DTI FA values and cognition

Exploratory analyses between FA values and cognitive measures for the group as a whole revealed a number of significant associations (Table 8). For the anterior commissure, lower FA values were significantly correlated with slower fine motor speed and dexterity with the dominant hand (Grooved pegboard; *r* = −0.338; *p* < 0.05). Lower FA values in this pathway were also associated with poorer sustained attention (RVP A′; *r* = 0.364; *p* < 0.005).

**Table 8:** Associations between FA values and cognitive measures

Cognitive domain	Measure	AC	LALIC	CC genu	RALIC
Visual scanning speed	TMT trails A	−0.051	−0.288	−0.157	−0.114
Cognitive set-shifting	TMT trails B	−0.274	−0.409**	−0.289	−0.182
Fine motor speed	GP–DH	−0.338*	−0.468**	−0.475**	−0.384*
Fine motor speed	GP–NDH	−0.145	−0.465**	−0.410**	−0.318*
Sustained attention	RVP A′	0.364*	0.403*	0.335*	0.094
Verbal learning	CVLT-II total recall	0.235	0.419**	0.206	0.343*
Immediate recall	CVLT-II SDFR	0.234	0.376*	0.065	0.242
Delayed recall	CVLT-II LDFR	0.222	0.416**	0.098	0.184
Verbal recognition	CVLT-II recognition	0.055	0.247	0.086	0.229
Concept shifting	IED EDS errors	−0.122	−0.053	−0.280	0.052
Concept formation	IED Pre-EDS errors	−0.080	−0.089	−0.317*	−0.003

Numbers represent Pearson correlations between cognitive scores and FA values

Significant correlation * *p* < 0.05, ** *p* < 0.01

*AC* anterior commissure, *LALIC* left anterior limb of the internal capsule, *CC* corpus callosum, *RALIC* right anterior limb of the internal capsule, *TMT* trail making test, *GP* grooved pegboard, *DH* dominant hand, *NDH* non-dominant hand, *RVP* rapid visual information processing, *CVLT-II* California verbal learning test-II, *SDFR* short-delay free recall, *LDFR* long-delay free recall, *IED* intra-extra dimensional shift, *EDS* extradimensional

For the left anterior limb of the internal capsule, lower FA values were significantly correlated with slower fine motor speed and dexterity bilaterally (Grooved Pegboard dominant hand: *r* = −0.468, *p* < 0.005; non-dominant hand: TMT Trail B; *r* = −0.409, *p* < 0.01). Higher FA values were associated better sustained attention (RVP A′; *r* = 0.403, *p* < 0.05). Further, higher FA values predicted better verbal learning (CVLT total recall; *r* = 0.419, *p* < 0.01) and memory, including after both short (CVLT Short Delay; *r* = 0.376, *p* = 0.017) and long delays (CVLT Long Delay; *r* = 0.416, *p* = 0.008).

For the right anterior limb of the internal capsule, FA values correlated negatively with both the dominant (*r* = −0.384, *p* = 0.014) and non-dominant (*r* = −0.318, *p* = 0.046) hand on the grooved pegboard task, reflecting slower fine motor speed and dexterity. Also, as FA scores increased, verbal learning performance was higher (CVLT total recall; *r* = 0.343, *p* = 0.03).

For the corpus callosum genu, FA values correlated negatively with both the dominant (*r* = −0.475, *p* = 0.002) and non-dominant (*r* = −0.410, *p* = 0.009) hand on the grooved pegboard task. Higher FA values predicted better sustained attention (RVP A′; *r* = 0.335, *p* = 0.037). Finally, there was a negative correlation between FA values and the IED pre-EDS errors score (*r* = −0.317, *p* = 0.046), reflecting poorer concept formation.

## Discussion

To our knowledge, this is the first study to examine cognitive function and white matter integrity in subjects with a history of heavy psychostimulant use and comorbid HIV infection, compared to healthy, matched control subjects. The main findings of the present study are that the patient group exhibited significantly lower white matter integrity, indicated by decreased FA values, in the four important white matter tracts of the anterior commissure, left and right anterior limb of the internal capsule, and the genu of the corpus callosum. With the exception of the anterior commissure, all tracts also showed a significant increase in MD and RD, suggesting that increases in axonal membrane permeability, for example by processes such as demyelination, may underlie the observed decrease in FA values. The patient group also displayed widespread neurocognitive impairment, which was significant in tasks that assess cognitive set-shifting, fine motor speed and verbal memory.

All subjects were recruited from the urban setting of the Downtown Eastside of Vancouver, which represents one of the poorest neighborhoods in Canada, with high rates of drug use, poverty, infectious disease and crime [31]. The mean age of the patient group was 37.5 years and length of HIV infection was 8.7 years. A majority of the patient group completed blood work and exhibited laboratory changes consistent with the literature on HIV [32], including decreased total white blood cell counts, decreased CD4 cell counts (immunodeficiency), increased CD8 cell counts (immunosuppression) and a lower CD4:CD8 ratio. While laboratory values in the patient group were lower than controls, most absolute values were close to accepted norms (http://aids.gov/index.html), suggesting that most subjects in the patient group were responding to treatment for HIV (although viral loads were not measured). Drug use was assessed over the 30 days prior to the interview. High rates of psychostimulant use were evident in all subjects in the patient group, which included powder cocaine, crack cocaine and amphetamines. Heroin and cannabis use were also common, indicating that drug use in this population is heterogeneous, and is consistent with simultaneous polysubstance use in an urban setting [33, 34]. The overall picture of the patient population is therefore that of individuals struggling not only with serious drug use and HIV infection, but potentially a host of additional medical co-morbidities. It is noteworthy that there was a significantly greater proportion of the control group that consumed alcohol than the patient group. Absolute amounts of alcohol consumed over the prior 30 days were not excessive in the control group (4.6 ± 5.7 units) so it seems unlikely that this could account for group differences in FA values, but this requires further verification.

The novel observation that subjects in the patient group had decreased white matter integrity in the four different forebrain pathways adds to our knowledge of the deleterious effects of comorbid HIV infection in heavy psychostimulant users on white matter in the brain [35]. Previous DTI studies in HIV positive subjects reported either a decrease in FA values [36] or no difference from controls [23, 37] in the genu of the corpus callosum, although [38] noted lower FA values only in HIV positive subjects with concurrent alcoholism. Other studies have reported decreases in FA values for the entire corpus callosum [21, 39], whereas the present study focused on a specific sub-region of this tract. Previous studies have not observed significant decreases in FA values of the anterior internal capsule in HIV positive subjects compared to controls [39–42], although [22] noted decreased FA values in the right *posterior* limb of the internal capsule. As such, our study represents the first evidence that the bilateral internal capsule pathways are affected in subjects with HIV infection. We are not aware of any studies that have measured FA values of the anterior commissure in HIV positive subjects.

The effects of psychostimulant drugs on white matter integrity have been the focus of several previous studies. The FA values in the genu of the corpus callosum were either decreased [12, 43] or not different [8, 44] from controls in cocaine users, while FA values were globally decreased compared to controls in current or former methamphetamine users [10, 11, 45]. We are not aware of prior reports of psychostimulant use on FA values for either the internal capsule or the anterior commissure. In this regard, the current observations are consistent with previous changes reported for the genu of the corpus callosum, and suggest that additional frontotemporal white matter pathways that control neurocognitive processes may be affected by comorbid HIV infection in heavy psychostimulant users.

In the present study, the patient group exhibited substantially lower performance across multiple cognitive domains compared to controls, which likely reflects in part the effects of HIV infection. While there is substantial variability in neurocognitive impairment in patients with HIV, deficits are commonly detected in the areas of speed of information processing [46], fine motor speed and dexterity [47], aspects of learning and memory (i.e., prospective memory, retrieval, etc.) [48] and multiple domains of executive functioning (i.e., mental flexibility, planning, etc.) [49]. Such deficits may be present even when antiviral medication is effective in controlling viral load [50]. Psychostimulant drugs, with extended use, can also significantly impair cognition [3]. Previous deficits in psychostimulant users have been reported with the same cognitive tasks that we used, including the Trail Making Test [51], grooved pegboard [52], CVLT [53], and CANTAB IED task [54].

Importantly, white matter integrity for the four tracts in the present study was significantly correlated with cognitive performance, indicating that decreased FA values and consequent loss of white matter integrity in the patient group may represent a physiological substrate for cognitive impairment in this population. Exploratory analyses suggested that all four tracts were each correlated with performance on multiple tasks, and significant correlations were consistent with the known neuroanatomy of specific tests.

Several previous studies have examined the impact of comorbid HIV infection and stimulant use on the brain with alternate MRI techniques. HIV positive cocaine users displayed impaired brain activation in an fMRI study with a delay discounting task [55]. Chang et al. [56] noted that the effects of HIV infection and chronic methamphetamine use were additive when they examined changes in brain metabolites in frontal white matter with MR spectroscopy, indicating that combined psychostimulant drug use and infection caused greater white matter damage. However, [57] noted that effects of both HIV infection and methamphetamine use were not additive in altering grey matter regional volumes, and in some basal ganglia sub-regions the two factors actually opposed each other. These two latter imaging studies suggest that white matter in the brain may be particularly vulnerable to additive effects of psychostimulant use and HIV infection. In the present study, the decreases in white matter integrity in the patient group included regions (left and right anterior limbs of the internal capsule, anterior commissure) not previously reported to be affected in HIV positive subjects without a confirmed history of psychostimulant abuse.

A potential limitation of this study is the absence of control groups that included recent psychostimulant users without HIV infection, or HIV positive subjects without psychostimulant use. This would have better enabled the study to demonstrate unambiguous effects of the drugs, or HIV, or an additive effect of both on the brain. However, the current patient group represents a marginalized population that is commonly encountered in urban settings characterized by high rates of poverty and antisocial behaviors such as crime [24, 33], and where drug use and HIV infection co-occur with extensive other medical comorbidities to affect brain function and structure. Potential comparison groups of HIV positive subjects without drug use, or psychostimulant users without infection (which would reflect different patterns of drug use) would likely represent fundamentally different populations from our study population, living under different and less challenging conditions. Parceling out the individual effects of HIV infection or psychostimulant use therefore presents a significant challenge, as it would be necessary in this population to control for the many other medical and environmental comorbidities that may be interacting to affect general physical health and the brain [25].

## Conclusions

In summary, the present study is the first report of white matter integrity, measured by DTI, in psychostimulant users with comorbid HIV infection. Four main frontotemporal white matter pathways were affected in this group, reflected by decreased FA values. Compared to controls, psychostimulant users with HIV infection displayed broad cognitive impairment, which significantly correlated with white matter integrity. Further study is required to better understand the influence of additional medical comorbidities, resulting largely from drug use, on white matter structure and cognition.

## Abbreviations

CANTAB: Cambridge neuropsychological test automated battery
CBC: complete blood count
CVLT-II: California verbal learning test II
DTI: diffusion tensor imaging
FA: fractional anisotropy
HIV: human immunodeficiency virus
IED: intra/extra dimensional shift
MRI: magnetic resonance imaging
NAART: North American adult reading test
ROI: region of interest
RVP: rapid visual information processing
TMT: trail making test
